# *Hematodinium* sp. and its bacteria-like endosymbiont in European brown shrimp (*Crangon crangon*)

**DOI:** 10.1186/2046-9063-8-24

**Published:** 2012-09-07

**Authors:** Grant D Stentiford, Kelly S Bateman, Hamish J Small, Michelle Pond, Anette Ungfors

**Affiliations:** 1 European Union Reference Laboratory for Crustacean Diseases, Centre for Environment, Fisheries and Aquaculture Science (Cefas), Barrack Road, Weymouth, Dorset, DT4 8UB, United Kingdom; 2 Virginia Institute of Marine Science, College of William and Mary, P.O. Box 1346, Gloucester Point, VA, 23062, USA; 3 Department of Biology and Environmental Sciences, Tjärnö, University of Gothenburg, Strömstad, SE, 452 96, Sweden

**Keywords:** ITS1, Phylogenetics, Dinoflagellate, Bacteria, Crustacean, Disease, Fishery

## Abstract

**Background:**

Parasitic dinoflagellates of the genus *Hematodinium* are significant pathogens affecting the global decapod crustacean fishery. Despite this, considerable knowledge gaps exist regarding the life history of the pathogen *in vivo*, and the role of free living life stages in transmission to naïve hosts.

**Results:**

In this study, we describe a novel disease in European brown shrimp (*Crangon crangon*) caused by infection with a parasitic dinoflagellate of the genus *Hematodinium*. This is the second example host within the Infraorder Caridea (shrimp) and significantly, the first description within the superfamily Crangonoidea. Based upon analysis of the rRNA gene (SSU) and spacers (ITS1), the parasite in *C. crangon* is the same as that previously described infecting *Nephrops norvegicus* and *Cancer pagurus* from European seas, and to the parasite infecting several other commercially important crab species in the Northern Hemisphere. The parasite is however distinct from the type species, *H. perezi*, found infecting type hosts (*Carcinus maenas* and *Liocarcinus depurator*) from nearby sites within Europe. Despite these similarities, the current study has also described for the first time, a bacteria-like endosymbiont within dinospore stages of the parasite infecting shrimp. The endosymbionts were either contained individually within electron lucent vacuoles within the parasite cell cytoplasm, or remained in direct contact with the parasite cytoplasm or in some cases, the nucleoplasm. In all of these cases, no apparent detrimental effects of colonization were observed within the parasite cell.

**Conclusions:**

The presence of bacteria-like endosymbionts within dinospore life stages presumes that the relationship between the dinoflagellate and the bacteria is extended beyond the period of liberation of spores from the infected host shrimp. In this context, a potential role of endosymbiosis in the survival of free-living stages of the parasite is possible. The finding offers a further intriguing insight into the life history of this enigmatic pathogen of marine crustacean hosts and highlights a potential for mixotrophy in the parasitic dinoflagellates contained within the genus *Hematodinium*.

## Background

Parasitic dinoflagellates of the genus *Hematodinium* are considered some of the most significant known pathogens affecting commercially exploited global decapod crustacean fisheries [1]. Recent reviews [1-3] indicate a wide host range, mainly across several superfamilies of the Brachyura (Portunoidea, Cancroidea, Calappoidea, Majoidea and Xanthoidea) but also the infraorders Anomura (subfamily Galatheoidea) and Astacidea (subfamily Nephropoidea). Recently, the host range has been further extended into the infraorder Caridea (subfamily Palaemonoidea) with the description of *Hematodinium*-like infections in farmed populations of the palaemonid ridgetail prawn *Exopalaemon carinicauda* in China [4]. In this first description of a *Hematodinium*-like dinoflagellate in prawns, the parasite was demonstrated to be likely the same as that causing similar infections in the Chinese swimming crab (*Portunus trituberculatus*) and mud crab (*Scylla serrata*) co-cultured in these farms [5,6], suggesting that the parasite may be a host generalist, a point elaborated in recent studies [7,8]. *Hematodinium*-like dinoflagellates have also been reported from a number of non-decapod crustaceans, namely members of the superorder Peracarida, order Amphipoda, although in most of these cases, molecular diagnosis and subsequent phylogenetic analysis was not carried out [9]. Small et al. [10] have demonstrated that at least one species, the crustacean carrion scavenging lysianassid amphipod *Orchomene nanus* was associated with a parasite with 98% identity (ITS1 rRNA partial sequence) to the *Hematodinium* sp. infecting *Nephrops norvegicus* from the same fishing grounds. Likewise Pagenkopp Lohan et al. [8] also report detecting *Hematodinium* DNA within Caprellid amphipods from estuaries from the east coast of the United States.

A study by Small et al. [11] focussed on characterizing the recently discovered type species, *Hematodinium perezi*, infecting one of the type hosts, *Liocarcinus depurator*, collected from near to the type location (English Channel, Europe). In that study, comparison of the ITS rRNA region sequences between the type species and others in GenBank revealed three distinct *H. perezi* genotypes infecting several portunid hosts (e.g. *Liocarcinus depurator*, *Callinectes sapidus*, *Portunus trituberculatus* and *Scylla serrata*). However, this is not an exclusive host grouping as both Xu et al. [4] and Pagenkopp Lohan et al. [8] have shown that *H. perezi* is capable of infecting other non-portunid hosts. Analysis of the parasites SSU rRNA gene and ITS regions by Small et al. [11] and others [12-14] suggests that a second, currently unnamed *Hematodinium* sp. infects a growing number of crustacean host species from the Northern Hemisphere. The latter includes well-documented disease outbreaks in the fisheries for *Nephrops norvegicus*[15] and *Cancer pagurus*[16,17] in Europe, and *Chionoecetes bairdi, Chionoecetes tanneri* and *Chionoecetes opilio* in Canada and Alaska [3].

In the current study, we describe a *Hematodinium* sp. parasite infection in wild *Crangon crangon* from the European fishery. For the first time, the parasite was shown to be colonised by bacteria-like endosymbiont that inhabited the cytoplasm, and occasionally the nucleoplasm of the parasite. Molecular phylogenetic analysis of the parasite from *C. crangon* suggests it to be the same as that infecting other decapod crustaceans from the Northern hemisphere (e.g. *Nephrops norvegicus, Chionoecetes opilio*, *Cancer pagurus, Carcinus maenas*).The findings are discussed in relation to the expanding range of known hosts to *Hematodinium* dinoflagellate infections and the nature of the relationship with the bacteria-like endosymbiont.

## Results

**Figure 1:**
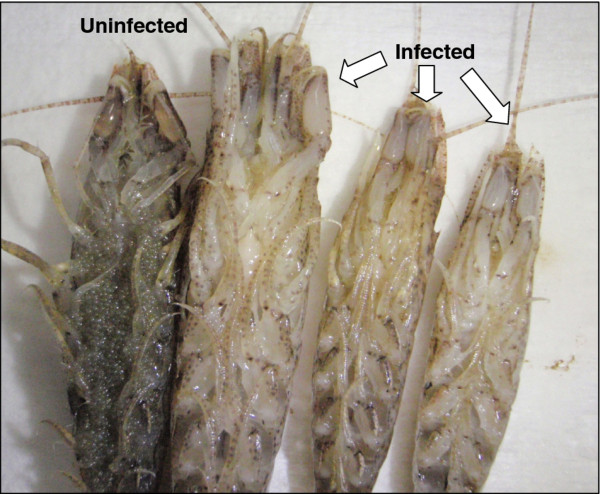
**External appearance of uninfected (left) and* Hematodinium*-infected (3 to right)* Crangon crangon*.** Infected hosts display a loss of transparency of the cuticle, particularly visible in the appendages. Loss of transparency is due to large numbers of parasite life stages within the haemolymph of infected shrimp.

**Figure 2:**
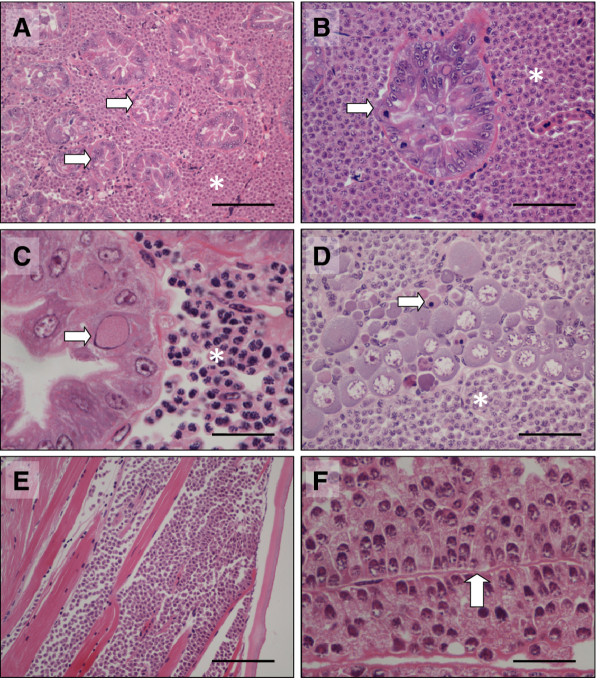
**Histopathology of* Hematodinium* sp. infection in* Crangon crangon*.** (**A**) Low power image showing distension of hepatopancreatic haemal sinuses with masses of *Hematodinium* sp. parasites (asterisk). Hepatopancreatic tubules are shrunken and are depleted of lipid inclusions (arrows). Scale 200 μm. (**B**). Single hepatopancreatic tubule (arrow) surrounded by masses of *Hematodinium* sp. parasitic life stages (asterisk) within the distended haemal sinuses. Scale 100 μm. (**C**) Hepatopancreatic tubule co-infected with *Crangon crangon* bacilliform virus (CcBV) (arrow) (Stentiford et al. 2004). *Hematodinium* sp. parasite life stages are seen within the haemal sinuses (asterisk) Scale 50 μm (**D**). Ovary, apparently arrested in pre-vitellogenic status. Oocytes do not contain vitellogenesis and oogonia are often apoptotic (arrow). Parasite life stages are in direct contact with oocytes (asterisk). Scale 100 μm. (**E**). Skeletal musculature within a walking appendage. Atrophy of muscle fibres is accompanied by colonisation of haemal spaces with masses of parasite cells. Scale 200 μm. (**F**). Attachment of filamentous trophonts to remnant basement membranes (arrow) within the atrophied skeletal musculature. Scale 25 μm. All images H&E histology.

Visual screening of a subsample of *C. crangon* from the Wash fishery in July 2010 indicated an apparent prevalence of 1.6% (8/480) of shrimp displaying abnormal opacity and lethargy (Figure 1). Affected shrimp exhibited a loss of carapace transparency and upon opening of the body cavity, contained milky, opaque haemolymph that did not clot. Histological analysis of tissues collected from affected shrimp revealed a systemic infection by a protistan pathogen with a limited, eosinophilic cytoplasm and a distinctive, basophilic nucleus containing condensed chromatin. In several cases, multi-nucleate plasmodial life stages were also observed, the cumulative effect being distension of the haemal sinuses (particularly within the hepatopancreas, where connective tissues and other inter-tubular tissues were apparently limited) (Figure 2A and B). The pathognomonic signs were typical of previous descriptions of *Hematodinium* spp. infections in crustacean hosts. Haemocyte encapsulation responses were not observed in any of the infected shrimp assessed though haemocytes were occasionally observed amongst the massed parasite cells. In all cases, the hepatopancreas of shrimp assessed using histopathology were co-infected with *Crangon crangon* bacilliform virus (*Cc*BV), previously described in European *C. crangon* populations [18,19]. In most shrimp, CcBV-infected epithelial cells were widely distributed throughout the organ (Figure 2C). In a manner consistent with *Hematodinium* sp. reported infecting other decapod hosts, the ovary of infected female shrimp was apparently arrested in pre-vitellogenic development. In addition, oogonia (early development stages of the ovarian lineage) were often apoptotic (Figure 2D). The skeletal musculature of infected shrimp was atrophied and contained enlarged haemal sinuses containing masses of parasite cells (Figure 2E). Filamentous trophont stages of the parasite were apparently attached to remnants of the basal membrane or sarcolemma of atrophied muscle fibres (Figure 2F). 

**Figure 3:**
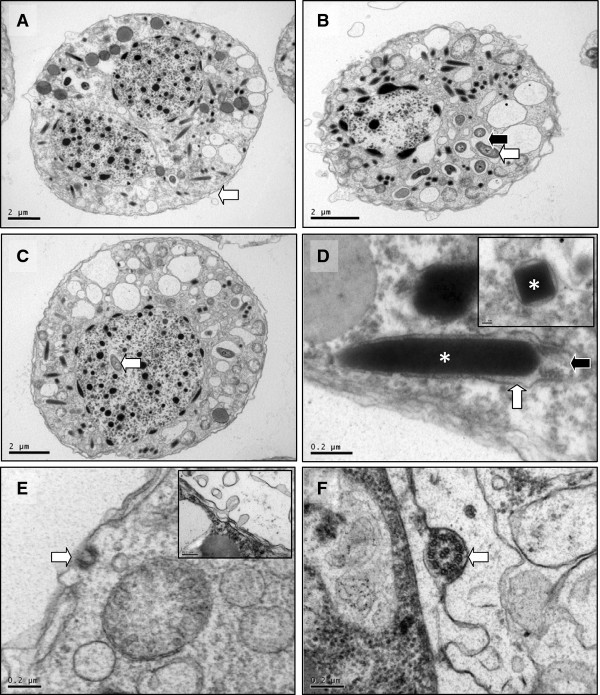
**Ultrastructure of trophont and spore stages of* Hematodinium* sp. from* Crangon crangon*.** (**A**) Bi-nucleate trophont containing trichocysts (T), mitochondria (M), vacuoles (V), Lipid Droplets (L) and a surrounding alveolar membrane (white arrow). (**B**) Dinospore with similar features to A. but with additional presence of numerous bacteria-like symbionts within the parasite cytoplasm (arrows). Bacteria-like symbionts appear to reside in an electron lucent vacuole (black arrow). (**C**) Dinospore with similar features to B but with additional presence of bacteria-like symbionts within the parasite nucleoplasm (arrow). (**D**) Detail of trichocyst within the parasite cytoplasm. The electron dense laminar core (asterisk in main image and inset) is surrounded by the trichocyst sac (white arrow) that terminates at the pusular sac (black arrow). (**E**) Detail of apicomplexan-like micropore (arrow) and blebbing of alveolar membrane surrounding the trophont (inset). (**F**) Flagella of dinospore stage displaying classic 9 + 2 arrangement of microtubules (arrow).

Transmission electron microscopy (TEM) revealed that the haemolymph and tissues of *C. crangon* harboured trophont (Figure 3A and C) and dinospore (Figure 3B) stages of a parasitic dinoflagellate parasite with features largely consistent with *Hematodinium* spp. previously described infecting other marine crustacean hosts. Trophonts contained trichocysts, mitochondria, large electron lucent vacuoles, and a distinctive surrounding alveolar membrane (Figure 3A). Trichocysts were defined by an electron dense, cuboid laminar core surrounded by a trichocyst sac, terminating with a distinctive pusular sac. In several instances, the pusular sac was closely opposed to the inner cell membrane of the parasite (Figure 3D). Structures resembling the micropores of apicomplexans were observed in several trophonts (Figure 3E). Spore stages were defined by the presence of flagella contained beneath the outer layer of the alveolar membrane and displaying a 9 + 2 arrangement of microtubules (Figure 3F).

**Figure 4:**
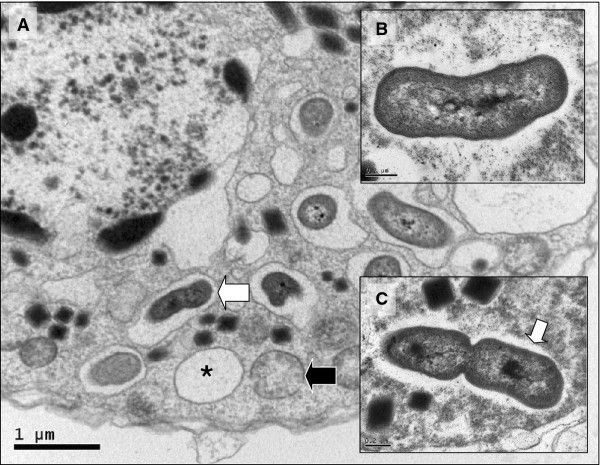
**Ultrastructure of bacterial symbiont within dinospore of* Hematodinium* sp. from* Crangon crangon*.** (**A**) bacteria-like symbionts generally occupied electron lucent vacuoles within the spore cytoplasm of the majority of individual parasites observed (white arrow). Empty electron lucent vacuoles (asterisk) and mitochondria (black arrows) were seen in close proximity to endosymbionts. (**B**) Single bacteria-like symbiont within the nucleoplasm of the parasite (as shown in Figure 3C). Note the apparent absence of the electron lucent vacuole generally seen surrounding bacteria-like symbionts occupying the cytoplasm. (**C**) Dividing endosymbiont within the cytoplasm of the parasite cell. Note in this case, the endosymbiont was not surrounded by a vacuolar membrane buy instead lay in direct contact with the host cytoplasm, albeit surrounded by an electron lucent halo (arrow).

In a manner not previously observed for the *Hematodinium* spp. dinoflagellates, dinospore life stages (Figure 3B and C), accommodated numerous bacteria-like endosymbionts (Figure 4A). Bacteria-like cells were either contained individually within electron lucent vacuoles within the parasite cell cytoplasm (Figure 4A; see also Figure 3B), or remained in direct contact with the parasite cytoplasm (Figure 4C) or in some cases, the nucleoplasm (Figure 4B; see also Figure 3C). In the case of those in direct contact with the cytoplasm, bacteria-like cells were occasionally observed undergoing fission (Figure 4C). In all cases where bacteria-like cells were observed within individual parasite cells, the ultrastructure of the parasite cell appeared otherwise normal when compared to parasite cells without bacteria-like endosymbionts. Although we cannot discount the presence of bacteria-like cells within trophonts, all observed cases were associated with dinospore life stages.

Analysis of the amplified rRNA fragments (containing the partial 3' end of the SSU gene and ITS1 region) confirmed that the parasite infecting *C. crangon* was a *Hematodinium* spp. Twenty two of the thirty clone sequences of the partial SSU sequence fragment (177 bp-immediately upstream of the SSU/ITS1 border) were identical, and the others differing from this common allele only by the presence of single nucleotide polymorphisms (SNP, n = 7) or by two SNPs (n = 1) within each clone sequence. BLAST analysis of the common SSU allele resulted in a 100% similarity to SSU sequences in GenBank for the *Hematodinium* sp. infecting *Nephrops norvegicus* (FJ844429)*, Chionoecetes angulatus* (FJ844426)*, Chionoecetes tanneri* (FJ844424)*, Chionoecetes opilio* (FJ844422)*, Chionoecetes bairdi* (FJ844417)*, Carcinus maenas* (EF675765)*, Cancer pagurus* (DQ871211)*, Pagurus bernhardus* (DQ871213), and other hosts. The ITS1 region sequences differed in length (between 327 and 335 bp), even among clone sequences generated from the same genomic DNA samples. This was due to a number of repetitive motifs previously described in the ITS regions of this *Hematodinium* sp. [13,20,21]. A number of SNPs were also observed. Eight of the 30 ITS1 sequences were identical. BLAST analysis of this common ITS1 allele resulted in a 100% similarity to ITS1 sequences in GenBank for the *Hematodinium* sp. infecting *Chionoecetes opilio* from Canada (FJ844422) and Greenland (FJ172640), *Lithodes couesi* (FJ844413), *Liocarcinus depurator* from Denmark (FJ172661), *Nephrops norvegicus* from Denmark (FJ172658) and Scotland (DQ871212), *Pagurus bernhardus* from Denmark (FJ172637) and Scotland (FJ495188), *Hyas araneus* from Greenland (FJ172644), and 99% similar to additional sequences from the above hosts and to *Cancer pagurus* from Scotland (DQ871211) and Ireland (EF031998). Three of the remaining ITS1 sequences were also identical and differed from those in GenBank from *Chionoecetes opilio*, *Lithodes couesi*, *Liocarcinus depurator*, *Nephrops norvegicus*, *Pagurus bernhardus*, and others by a single base deletion. The remaining 19 ITS1 sequences were unique, however these differed from those in GenBank by a single SNP (n = 9), or by a combination of SNPs and insertions/deletions of repetitive motifs. The partial SSU and complete ITS1 rRNA sequences from the *Hematodinium* sp. infecting *C. crangon* have been deposited in GenBank with accession numbers JX499154- JX499183.

## Discussion

**Figure 5:**
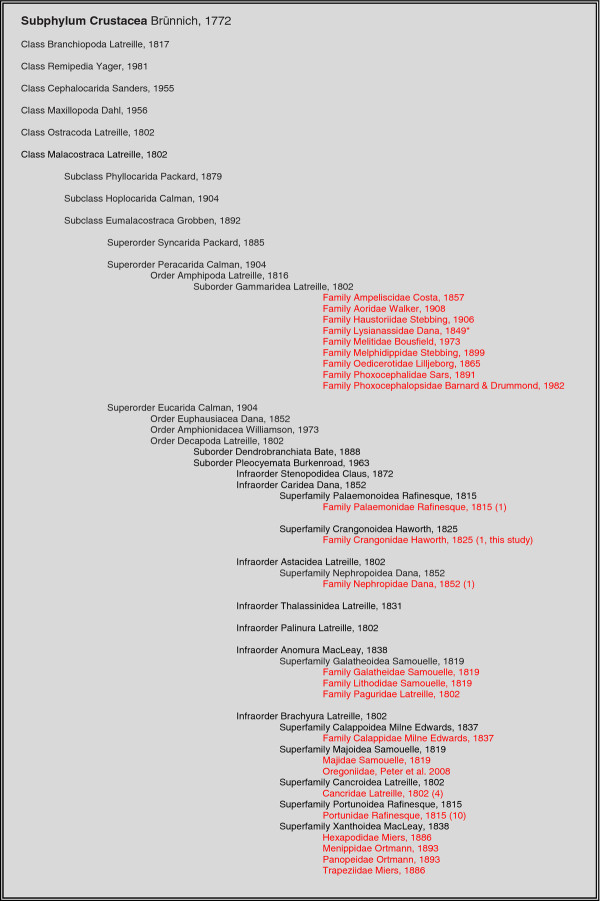
**Summary of host range to* Hematodinium* dinoflagellate infections across the Class Malacostraca of the Subphylum Crustacea.** Taxa with representative host species are shown in red. Numbers in parentheses refer to the approximate number of described hosts within a specific taxonomic group at the time of writing.

Parasitic dinoflagellates comprising the genus *Hematodinium* have been described in over 40 crustacean host taxa [1]. These descriptions include infections of hosts across two Superorders (Pericarida and Eucarida) of the Subclass Eumalacostraca (Class Malacostraca). Within the Eucarida, all host taxa described to date reside within the Order Decapoda, Suborder Pleocyemata. Within the Pleocyemata, *Hematodinium* sp. parasites have been described infecting hosts from the Infraorders Caridea, Astacidea, Anomura and Brachyura. To date, the majority of descriptions relate to hosts residing within the Brachyura, possibly due to the fact that the large, commercially fished crab species are contained within this Infraorder and have thus received most attention as part of field surveys. A summary of host range across the Class Malacostraca is provided in Figure 5. In addition to the widely divergent (and likely under-reported) host range, infection by parasitic dinoflagellates of the genus *Hematodinium* have been reported in crustacean from across the globe, encompassing host species from Australian, European, North American, Canadian, Alaskan, Russian and Chinese waters [1]. The pronounced pathology associated with patent disease has led to marketing and fishery consequences for infected hosts and their populations [2]. In this context, the disease associated with *Hematodinium* sp. infection is considered as a key health issue affecting the sustainability of the global crustacean fishery [22]. 

Despite this diversity in host, geographic and habitat range, considerable evidence now exists to suggest that two different species of *Hematodinium* infect the majority of known hosts, at least in the Northern Hemisphere [1,11,13]. Data relating to sequencing of the rRNA genes (SSU) and spacers (ITS1) support the separation of the type species (*H. perezi*) from a currently unnamed second species (referred to as *Hematodinium* sp. in several recent publications [11-14,20,23]. Based on the rRNA sequences obtained from infected hosts, the parasite described herein infecting brown shrimp (*C. crangon*) from European waters also falls into the latter (*Hematodinium* sp.), despite the relatively close geographic existence between the field site utilized here and that of the recent description of the type species, *H. perezi* in *L. depurator*[11]. Recent studies have also suggested some level of host specificity within the *Hematodinium* sp. infecting hosts from the North Atlantic. Hamilton et al. [21] describe three distinct genotypic groups (based on *Hematodinium* sp ITS1 sequences) that correspond to hosts species rather than geographic location. However, in the current study the major *Hematodinium* sp. ITS1 allele from *C. crangon* was identical to those in GenBank from multiple hosts, including *C. opilio*, *L. depurator*, *N. norvegicus*, *P. bernhardus*, and others. Likewise, the minor and unique ITS1 alleles were also identical to ITS sequences from multiple hosts, or differed by a combination of SNPs and repetitive elements, respectively, suggesting no relationship between host species. An additional confounding factor is that the majority of recent studies reporting rRNA sequences from the *Hematodinium* sp. have utilized direct sequencing techniques [7,13,21]. Therefore, the ITS1 sequences deposited in GenBank may not represent the true allelic diversity of the *Hematodinium* sp. in a particular host.

The current description of *Hematodinium* sp. infection in *Crangon crangon* provides a further example host within the Infraorder Caridea (shrimp) and significantly, the first description within the superfamily Crangonoidea. Previously, *Hematodinium perezi* has been reported from a member of the superfamily Palaemonoidea (*Exopalaemon carinicauda*), co-cultured with the brachyuran crabs (*Portunus trituberculatus* and *S. Serrata*) in China [1,4]. The infection of *P. trituberculatus*, *S. Serrata*, and *E*. *carinicauda* by *H. perezi*[4-6] provided the first examples of *H. perezi* as a potential problem in the global crustacean aquaculture industry, and suggests that this pathogen is capable of infecting novel species under polyculture conditions. This feature further reinforces the likelihood for low host specificity in this parasite genus [7,8,13,20]. Taken together, these data suggest that both known types (*H. perezi* and *Hematodinium* sp.) can infect different hosts residing in relative close proximity to one another, at least in waters including and adjacent to, the English Channel.

In the current study, we also report the presence of a bacteria-like endosymbiont within the *Hematodinium* sp. parasite infecting *C. crangon*. Despite numerous published studies which have provided detailed ultrastructural data relating to infections by both *H. perezi*[11] and *Hematodinium* sp. [6,15,22,24-27], similar endosymbiotic associations have not previously been observed. The bacteria-like endosymbionts occurred singly within electron lucent vacuoles, or in direct contact with the dinospore cytoplasm. In rare cases, they were also observed in direct contact with the dinospore nucleoplasm and also apparently undergoing fission. In no cases were obvious detrimental effects of colonization observed within the parasite cell. The presence of bacteria-like endosymbionts within spore life stages presumes that the endosymbiont may remain with the spore following its presumed liberation from the infected host shrimp. Ultrastructural analysis of free-living life stages of *Hematodinium* liberated to the water column following sporulation in crustaceans hosts [2,28] may elucidate whether bacterial endosymbionts play a role in dinospore survival in open water.

Endosymbiotic relationships such as that described here entail complementation of the host’s metabolic capacity by that of the endosymbiont, possibly enabling the host to exist in previously unsuitable environments [29]. Several examples exist whereby dinoflagellates have been shown to harbour a range of prokaryotic and eukaryotic endosymbionts including alga [30,31] and bacteria, both within their cytoplasm [32] and even their nucleoplasm [33]. The most common forms of association relate to photosynthesis, nitrogen fixation or methanogenesis, although in the majority of cases, little more than a morphological description of the association is available [29]. Wilcox [32] proposes that although the nature of the association between the dinoflagellate and the bacteria is often enigmatic, both appear healthy, suggesting a stable relationship rather than a pathogenic one. Schweikert and Meyer [34], working on the freshwater dinoflagellate *Peridinium cincture* described colonisation by two previously undetected bacterial symbionts belonging to the eubacterial α- and γ-subgroups of proteobacteria. In this case, the bacterial symbionts maintained a stable association with the dinoflagellate in culture for over 2.5 years. In such instances, maintenance is suggestive of some benefit for the host cell. Regardless of the nature of the relationship between the bacteria-like endosymbiont and *Hematodinium*, the finding is the first of its kind in the Order Syndiniales and may suggest an element of mixotrophy in the life cycle of *Hematodinium*[35].

In other scenarios, algae (including the dinoflagellates) have been shown to be afflicted by a large number of bacterial pathogens which cause disease and impacts on fundamental properties of aquatic ecosystems [36]. Bacterial pathogens identified in alga to date include the Gram-negative genera *Alteromonas*, *Cytophaga*, *Flavobacterium*, *Pseudomonas*, *Pseudoalteromonas*, *Saprospira* and *Vibrio*. In addition to pathogenic effects on reef-building coralline algae, closely related bacteria also co-occur with pelagic bloom-forming microalgae such as dinoflagellates, and display potent algicidal properties [36]. These properties have even been exploited for mitigating the red tides formed by blooms of these algae [37].

## Conclusions

The incorporation of bacteria by heterotrophic eukaryotes has been implicated in the establishment of reduced endosymbionts, functioning as cellular organelles (e.g. for photosynthesis) within the host cell. Genomic reduction within the endosymbiont which accompanies this acquisition then blurs the distinction between ‘endosymbiont’ and ‘organelle’ [29]. Clearly in the case of *Hematodinium*, we know little of the nature of the association between the bacteria-like endosymbiont and the parasite in crangonid shrimp. Nevertheless, the finding offers a further intriguing insight into the life history of this enigmatic pathogen. Further effort is now required to identify the endosymbiont and to investigate the nature of its relationship with *Hematodinium*.

## Methods

### Collection of specimens and processing for histology and electron microscopy

Brown shrimp (*Crangon crangon*) were collected in July 2010 during the annual Clean Seas Environmental Monitoring Programme (CSEMP) survey of United Kingdom marine waters. Shrimp were specifically collected from sites within the Wash, North Sea (53.1417 N, 0.555 W). Upon capture, animals were placed live onto large trays for visual health assessments. During sorting, abnormal looking animals with an opaque carapace and apparently milky consistency of the haemolymph and underlying tissues were removed from the catch for processing (Figure 1). Representative examples of externally normal shrimp and those displaying signs of opacity were chilled on ice prior to dissection.

For histopathology, the hepatopancreas, gills, heart, midgut, gonad and skeletal muscles were dissected from each specimen. Excised samples were placed immediately into Davidson’s seawater fixative. Fixation was allowed to proceed for 24 h before samples were transferred to 70% industrial methylated spirit for storage prior to processing. Fixed samples were processed to wax in a vacuum infiltration processor using standard protocols. Sections were cut at a thickness of 3 to 5 μm on a rotary microtome and were mounted onto glass slides before staining with haematoxylin and eosin (HE). Stained sections were analysed by light microscopy (Nikon Eclipse E800) and digital images were taken using the Lucia™ Screen Measurement System (Nikon).

For electron microscopy, 2 mm^3^ blocks of skeletal muscle and hepatopancreas were fixed in a solution containing 2.5% glutaraldehyde in 0.1 M sodium cacodylate buffer (pH 7.4), for 2 h at room temperature prior to rinsing in 0.1 M sodium cacodylate buffer with 1.75% sodium chloride (pH 7.4) and post-fixation in 1% osmium tetroxide in 0.1 M sodium cacodylate buffer for 1 h at 4°C. Specimens were washed in three changes of 0.1 M sodium cacodylate buffer and dehydrated through a graded acetone series. Specimens were embedded in Agar 100 epoxy resin (Agar Scientific, Agar 100 pre-mix kit medium) and polymerised overnight at 60°C in an oven. Semi-thin (1–2 μm) sections were stained with Toluidine Blue for viewing with a light microscope to identify suitable target areas. Ultrathin sections (70–90 nm) of target areas were mounted on uncoated copper grids and stained with 2% aqueous uranyl acetate and Reynolds’ lead citrate. Grids were examined using a JEOL JEM 1210 transmission electron microscope and digital images captured using a Gatan Erlangshen ES500W camera and Gatan Digital Micrograph™ software.

For molecular analysis, gill and hepatopancreas samples corresponding to those regions sampled for histology and electron microscopy were dissected and placed directly into 100% ethanol. Genomic DNA was subsequently extracted from these tissues from *C. crangon* histologically identified as having *Hematodinium* sp. Infections. Tissue was weighed and homogenized in G2 buffer and proteinase K (Qiagen), using a Fastprep™ tissue disruptor (MP Biomedicals), to give a 10%w/v. DNA was extracted from a 50 μl volume of the homogenate using the EZ1 DNA Tissue kit (Qiagen) and the BioRobot EZ1 workstation (Qiagen), following the manufacturer’s protocol. DNA was eluted in 50 μl buffer and stored at −20°C prior to use in PCR assays. The 3' end of the SSU rRNA gene and the first internal transcribed spacer (ITS1) region of the *Hematodinium* sp. infecting *C. crangon* were amplified from six genomic DNA samples (isolated from separate shrimp) using the forward and reverse primer pairs A (5'-GTTCCCCTTGAACGAGGAATTC-3') and B (5'- CGCATTTCGCTGCGTTCTTC-3') from Hudson and Adlard (1994). Amplification reactions were carried out in a DNA Engine Tetrad 2 (MJ Research) and contained approximately 100 ng genomic DNA, 1x Green GoTaq Flexi Buffer (Promega), 2.5 mM MgCl_2_, 0.25 mM of each dNTP, 0.5 μM of each primer, 1.25 units GoTaq DNA polymerase and molecular grade water to a final volume of 50 μl. Reactions were overlaid with oil and 30 cycles of the following carried out: denaturation at 94°C for 1 min; primer annealing at 55°C for 30 s; extension at 72°C for 90 s, followed by a final 7 min extension.

PCR products were resolved on a 2% (w/v) agarose/TAE (40 mM Tris acetate, pH7.2, 1 mM EDTA) gel containing 0.625 mg/ml ethidium bromide and viewed under UV light. Gel fragments of approximately 680 bp were excised and purified using the Wizard® SV Gel and PCR Clean-Up System (Promega). Purified amplification products were ligated into the pGEM®-T cloning vector (Promega) and plasmid inserts were bi-directionally sequenced using the amplification primers (A and B) and BigDye® Terminator v3.1 Cycle Sequencing Kit (Applied Biosystems), following the manufacturer’s protocol. Sequencing reactions were electrophoresed on an ABI 3130xl Genetic Analyzer (Applied Biosystems). For each of the six genomic *C. crangon* DNA samples amplified, five clones were sequenced bi-directionally. *Hematodinium spp.* sequences were constructed from the forward and reverse sequencing reactions using Sequencher (version 4.1.4). Primer sequences and regions of poor resolution were removed from the 5´ and 3´regions, and the borders of flanking SSU and 5.8S RNA genes identified by comparison with sequences from previous studies [12,14]. The resulting partial SSU and ITS1 sequences obtained were compared for similarity to other *Hematodinium* spp. sequences in GenBank using the Basic Local Alignment Search Tool (BLAST) function [38]. The partial SSU and complete ITS1 rRNA sequences from the *Hematodinium* sp. infecting *C. crangon* have been deposited in GenBank with accession numbers JX499154- JX499183.

## Competing interests

The authors declare that they have no competing interests.

## Authors’ contributions

GDS was responsible for initial discovery of the pathogen, pathology and ultrastructural work and initial drafting of the manuscript. KSB was responsible for initial fieldwork, pathology and ultrastructural analysis of the pathogen. MP and AU were responsible for molecular diagnostics (PCR and sequencing) and for providing appropriate text to the manuscript. HJS was responsible for overseeing molecular phylogenetic assessments and for providing text to the manuscript. All authors read and approved the final manuscript.

## Authors’ information

GDS is Director of the European Union Reference Laboratory for Crustacean Diseases.
